# Extracellular vesicles in atherosclerosis cardiovascular disease: emerging roles and mechanisms

**DOI:** 10.3389/fcvm.2025.1611557

**Published:** 2025-06-24

**Authors:** Haoxuan Deng, Wei Qiu, Yunyan Zhang, Junyi Hua

**Affiliations:** Second Affiliated Hospital, Zhejiang Chinese Medical University, Hangzhou, Zhejiang, China

**Keywords:** extracellular vesicles, atherosclerosis, cardiovascular disease, inflammatory response, intercellular communication

## Abstract

The pathogenesis of atherosclerotic cardiovascular disease is complex, involving multiple cell types and biological processes. Extracellular Vesicles (EVs) are small, cell-derived particles increasingly recognized for their role in cardiovascular diseases. EVs are believed to play key roles in this context by promoting inflammation, regulating intercellular communication, and influencing lipid metabolism. As a crucial mediators of cell communication, EVs contribute to both the progression of atherosclerosis (AS) and plaques stability. Although research on the role of EVs in AS and the role of biomarkers or drug carriers in clinical practice has been expanding, several challenges remain for clinical applications, including the lack of specific therapeutic targets for EVs, flaws in the separation and purification processes, and limited clinical trial data on their safety. This review provides a comprehensive overview of the function of EVs in AS and recent advances in their diagnostic and therapeutic potential, aiming to inform future clinical applications.

## 1 Introduction

Atherosclerotic cardiovascular disease (ASCVD) is one of the leading causes of death and disability worldwide, which both incidence and mortality continuing to rise (1). ASCVD can also lead to severe complications such as heart failure, arrhythmias, and renal failure (2), affecting the patients' quality of life and imposing a substantial burden on healthcare systems. Currently, lowering LDL-C levels remains the cornerstone of ASCVD prevention and treatment. However, in clinical practice, many patients remain at high risk of cardiovascular events despite achieving optimal LDL-C control (3). Even with a combination of pharmacotherapy, lifestyle interventions, and surgical interventions, some patients continue to exhibit residual cardiovascular risk (4).

Extracellular Vesicles (EVs), as important mediators of intercellular communication, have garnered increasing interest in biomedical research. While early studies mainly focused on their biological characteristics (5), more recent investigations have highlighted their emerging potential in clinical diagnosis and therapy (6, 7). EVs derived from endothelial cells, platelets, vascular smooth muscle cells, monocytes, and macrophages are implicated in various mechanisms involved in atherosclerosis (AS), including modulation of endothelial cell function, promotion of inflammatory responses, platelet activation and vascular remodeling (8–10). Moreover, specific molecules carried by EVs not only reflect the status of their cell of origin but can also serve as biomarkers for the early diagnosis and monitoring of ASCVD (11, 12). From a therapeutic perspective, EVs are considered ideal drug delivery vehicles due to their inherent biocompatibility and targeting capabilities (13, 14), enabling efficient delivery of therapeutic agents to diseased sites. Engineered EVs, derived from natural ones, may further enhance targeting capability and therapeutic efficacy (15), offering innovative strategies for ASCVD treatment.

This review aims to elucidate the role of EVs in ASCVD pathogenesis and progression, providing new insights for their clinical application and laying the foundation for future targeted therapies.

## 2 Classification and characterization of extracellular vesicles

EVs are small, membrane-bound vesicles secreted by cells that play a crucial role in intercellular communication and material exchange. Based on their origin, size, and biological characteristics, EVs are classified into the exosomes, the microvesicles, and the apoptotic bodies (16).

### 2.1 Exosome

Exosomes are small EVs, typically ranging from 30 to 150 nanometers in diameter, surrounded by a lipid bilayer. They contain various bioactive substances such as proteins, lipids, and RNA (including miRNA and mRNA) (17). Surface proteins such as cluster of differentiation 9 (CD9), cluster of differentiation (CD63), and cluster of differentiation (CD81) are often present, facilitating their formation, release, and recognition (18). Additionally, exosomes lipid composition can influence their binding to target cells and uptake efficiency (19), making them valuable as drug delivery vehicles.

The biosynthesis and release process of exosomes is complex. Exosome biogenesis involves the invagination of the cell membrane to form endosomes, which mature into multivesicular bodies (MVBs). Within MVBs, intraluminal vesicles form through inward budding and are release as exosomes when MVBs fuse with the plasma membrane (20). Exosomes play a vital role in intercellular communication by transporting signaling molecules, miRNA, mRNA, and proteins, and protein to neighboring or distant cells, influencing various cellular processes such as cell growth, differentiation, movement, and death (21).

### 2.2 Microvesicles

Microvesicles (MVs) are EVs secreted by cells, typically ranging from 100 to 1,000 nanometers in diameter. They contents mRNA, miRNA, proteins, and lipids, which can influence target cells function (22). MVs form through the budding of the cell membrane in response to specific stimuli, with calcium influx, cytoskeletal changes, and membrane movement regulating the process (23).

MVs are efficient in cell-to-cell communication, primarily by transporting bioactive substances and binding on target cells, activating downstream signaling pathways (22). In immune responses, MVs play a dual role, promoting immune responses while potentially leading to immune suppression. MVs can promote immuity by carrying tumor-specific antigens that activat dendritic cells and enhance anti-tumor responses (24), but they can also suppress immunity (25). MVs from tumor cells can carry immunosuppressive factors that inhibit T cell function, contributing to immune evasion in the tumor's microenvironment (17). Additionally, some MVs influence macrophages differentiation, carrying cytokine that promote an anti-inflammatory M2 phenotype (26), aiding tissue repair and regeneration (27).

### 2.3 Apoptotic bodies

Apoptotic bodies are membrane-bound vesicles formed during apoptosis, typically measuring 5–10 micrometers in diameter. They arise from the breakdown of the cell membrane during the final stages of programmed cell death (28). These vehicles contain a complex mix of components, including membrane proteins, cytoplasmic contents, and organelles fragments (28). The membrane may carry proteins such as adhesion and transport proteins (29), while the cytoplasmic components include enzymes, RNA, and small molecules (30). Upon release, these components can influence neighboring cells, potentially promoting either apoptosis or proliferation (30). Additionally, residual organelles like mitochondria and the endoplasmic reticulum can release pro-inflammatory factors during apoptosis, further affecting surrounding cells (31).

The formation of apoptotic bodies involves distinct morphological changes, such as cell shrinkage, chromatin condensation, and membrane blebbing (32, 33). Besides facilitating the clearance of cellular debris, apoptosis bodies may also modulate autophagy and apoptosis of adjacent cells through their contents, forming a feedback regulation mechanism (34). In the immune responses, apoptotic bodies enhance the phagocytic activity of macrophages and dendritic cells by exposing signals like phosphatidylserine, promoting the clearance of dying cells and preventing autoimmune responses triggered by self-antigens (35).

## 3 The role of EVs in the pathogenesis of ASCVD

### 3.1 Evs in endothelial cell activation and dysfunction

**Figure 1:**
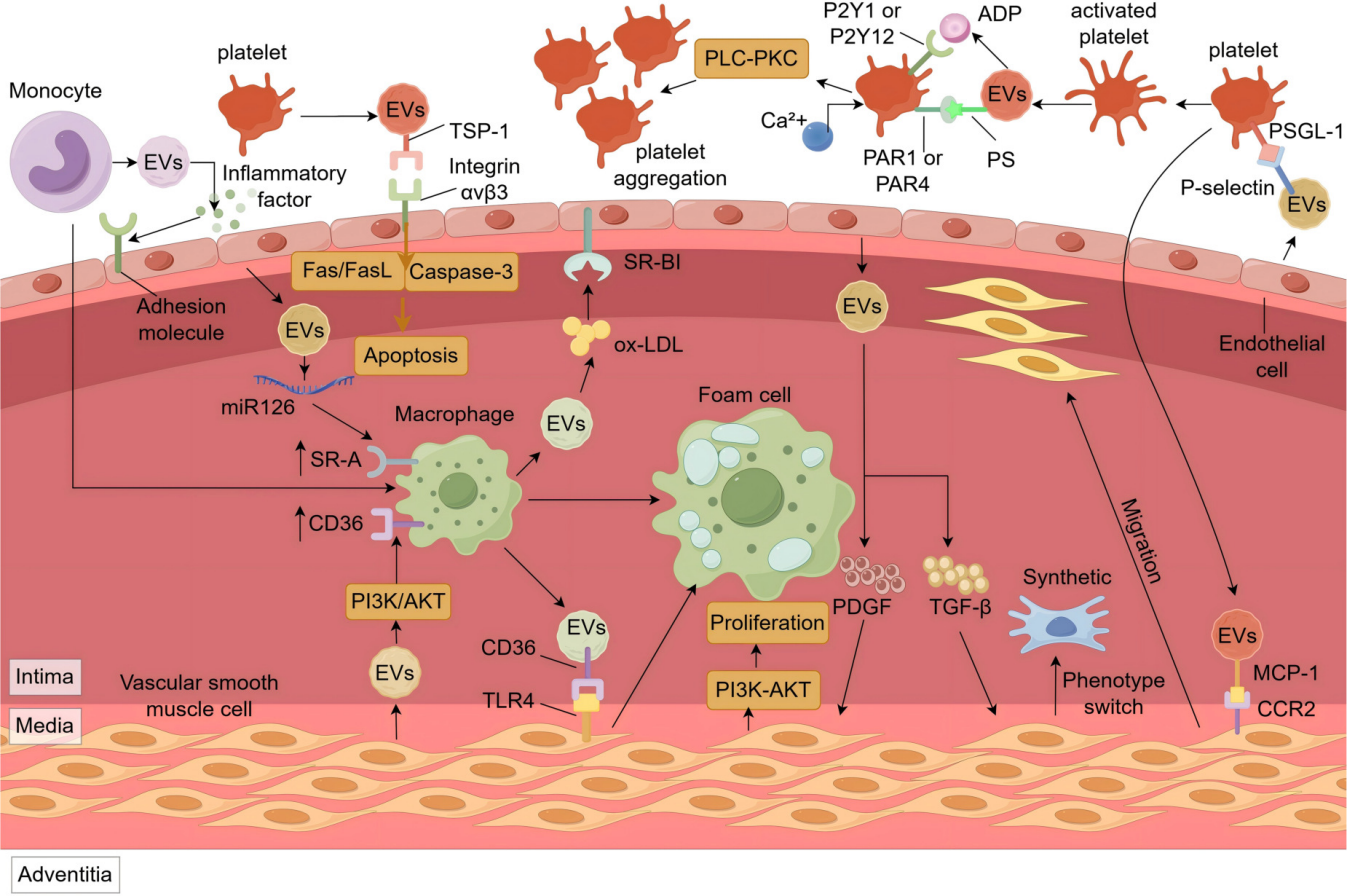
The role of EVs in the pathogenesis of ASCVD. M-EVs promote endothelial adhesion molecule expression via inflammatory ligands. P-EVs induce endothelial apoptosis through TSP-1/αvβ3-mediated Fas/FasL signaling and ROS/caspase-3 activation. Cholesterol-rich EVs from macrophages enter endothelial cells via SR-BI or stimulate ox-LDL uptake in SMCs via CD36/TLR4. Endothelial EVs with miR-126 and SMC-EVs with α-SMA/miR-21 upregulate SR-A and CD36 in macrophages, promoting foam cell formation. PDGF and TGF-β in endothelial EVs drive SMC proliferation (PI3K-Akt) and phenotype switching. MCP-1 in P-EVs promotes SMC migration via CCR2. P-EVs activate platelets via PS-receptor interaction, thrombin/PAR1/4, and ADP/P2Y1/12 signaling, increasing Ca^2+^ and promoting aggregation. EVs, Extracellular Vesicles; SR-A, scavenger receptor class A; CD-36, cluster of differentiation 14; Fas/FasL, tumor necrosis factor receptor superfamily member 6/tumor necrosis factor ligand superfamily member 6; TSP-1, thrombus proteins like thrombospondin-1; PI3K/Akt, phosphatidylinositol 3-Kinase/protein kinase B; SR-BI, scavenger receptor class B type I; TLR4, toll-like receptor 4; PLC-PKC, phospholipase C-protein kinase C; P2Y1, P2Y purinoceptor 1; P2Y12, P2Y purinoceptor 12; PAR1, protease activated receptor 1; PAR4, protease activated receptor 4; PS, phosphatidylserine; PDGF, platelet-derived growth factor; TGF-β, transforming growth factor beta; PSGL-1, P-selectin glycoprotein ligand-1; MCP-1, monocyte chemoattractant protein-1; CCR2, C-C motif chemokine receptor 2.

EVs can adhere to and interact with endothelial cells through various ligands like P-selectin (36), αvβ3, and α4β1 integrins (37). Upon recognition and binding, endothelial cells internalize EVs through mechanisms including endocytosis, membrane fusion, and phagocytosis (38). This uptake contributes to the progress and exacerbation of AS by promoting inflammation, apoptosis, and endothelial dysfunction (Figure 1).

Monocyte- and macrophage-derived EVs can carry pro-inflammatory molecules such as tumor necrosis factor-alpha (TNF-α), interleukin-1 beta (IL-1β), and IL-6 (39–42). Under inflammatory conditions or upon ox-LDL stimulation, these EVs transfer their cargo to endothelial cells, where ligands such as lipopolysaccharides engage toll-like receptor 4 (TLR4), activating the NF-κB signaling pathway (43, 44). Once activated, NF-κB translocates to the nucleus, inducing the expression of cell adhesion molecules, chemokines, and additional cytokines (42, 45). This persistent signaling forms a self-amplifying loop that facilitates leukocyte adhesion, exacerbates vascular inflammation, and accelerates the progression of atherosclerotic plaques.

EVs can contribute to endothelial apoptosis (Figure 1), thereby compromising vascular endothelium. Studies have shown that platelet-derived EVs (P-EVs) are significantly elevated in patients with acute coronary syndrome (46). These P-EVs can induce endothelial apoptosis via the mitochondrial pathway (47). On the one hand, surface proteins on P-EVs, such as thrombospondin-1 (TSP-1), can bind to integrin αvβ3 on endothelial cells, triggering the tumor necrosis factor receptor superfamily member 6/tumor necrosis factor ligand superfamily member 6(Fas/FasL) signaling cascade and mitochondrial damage (48). On the other hand, P-EVs can increase intracellular reactive oxygen species (ROS) levels (47, 49), leading to reduced mitochondrial membrane potential (50), release of cytochrome c, activation of caspase-9, and ultimately caspase-3 activation, initiating apoptosis (51). Apoptosis endothelial cells compromised the vascular barrier, facilitating the infiltration of lipids and inflammatory cells into the subendothelial space, thereby promoting plaque instability and rupture. However, EVs derived from pulmonary microvascular endothelial cells have been found to enhance the integrity of the endothelial barrier by transferring miR-125b-5p and inhibiting cell apoptosis (52), providing a direction for the treatment of ASCVD.

### 3.2 Evs are involved in foam cell formation

EVs are not only important mediators of intercellular communication but also regulate multiple aspects of lipid metabolism through various mechanisms, thereby promoting foam cells formation and influencing the initiation and progresses of AS (Figure 1).

The promotion of cholesterol synthesis by EVs primarily involves the sterol regulatory element-binding protein 2 (SREBP-2) pathway. EVs derived from cardiomyocytes and endothelial cells (53, 54) containing miR-9-5p and oxidized lipids, such as 7-ketocholesterol, can activate this pathway. Specifically, miR-9-5p inhibits Insig1 (55), thereby relieving its suppression of SREBP cleavage-activating protein and allowing for the activation and nuclear translocation of SREBP-2 (56); oxidized lipids can also induce endoplasmic reticulum stress, promoting the cleavage of SREBP-2 into its active forms (57). As a key transcription factor, SREBP-2 enhances the expression of 3-hydroxy-3-methylglutaryl-CoA reductase and squalene epoxidase upon nuclear entry (58), thereby boosting cholesterol synthesis (59). Simultaneously, it upregulates LDL-R expression (60), promoting uptake. Moreover, SREBP-2 increases proprotein convertase subtilisin/kexin type 9(PCSK9) expression, accelerating LDLR degradation and leading to elevated LDL-C levels, thereby increasing intracellular cholesterol concentrations.

Regarding cholesterol uptake, EVs exert influence via two primary mechanisms: directly cholesterol uptake and modulation of cell surface receptors involved in uptake. Firstly, EVs can directly transport cholesterol; macrophages under cholesterol overload conditions encapsulate cholesterol into EVs via transporters such as ATP-binding cassette sub-family A member 1 (ABCA1) and scavenger receptor class B type I (SR-BI) (61). These EVs can taken up by endothelial cells specific receptors (like SR-BI), or interact with cluster of differentiation 36 (CD36) carried on the EVs surface (62), which in turn binds to TLR4 on the smooth muscle cells, enhancing ox-LDL uptake (63, 64). This promotes droplet accumulation in endothelial cells (65) and activates the NLR family pyrin domain containing 3 (NLRP3) inflammasome in smooth muscle cells (66), accelerating their transformation into foam cells. Secondly, EVs can modulate ox-LDL receptor expression. Endothelial cell-derived EVs, once taken up by macrophages, can release miR-126, which inhibits sprouty-related EVH1 domain-containing protein 1 (Spred-1) expression (67), thereby activating the rat sarcoma/extracellular signal-regulated kinase (RAS-ERK) signaling pathway (68). The activation enhances scavenger receptor class A (SR-A) and CD36 expression (69), promoting ox-LDL uptake by macrophage (70). Meanwhile, smooth muscle cell-derived EVs can upregulate SR-A and CD36 in macrophages via activation of the phosphatidylinositol 3-kinase/protein kinase B (PI3K-Akt) signaling pathway (71), further promoting foam cell formation (72).

EVs also inhibit intracellular cholesterol efflux mainly by suppressing cholesterol transporters proteins, ABCA1 and ATP-binding cassette sub-family G member 1 (ABCG1), and by inhibiting macrophage autophagy. Under pathological conditions such as obesity, adipocyte-derived EVs enriched wirh fatty acid binding protein 4 inhibit the peroxisome proliferator-activated receptor gamma signaling pathway upon uptake by macrophages, resulting in downregulation of liver X receptor alpha(LXRα) (73, 74). Consequently, LXRα-mediated transcription of ABCA1 and ABCG1 is reduced, impairing cholesterol efflux (75). Furthermore, EVs enriched in miR-155-released by macrophages upon TNF-αstimulation (76), can suppress LXRαexpression, and directly inhibit translation of autophagy-related protein 5 and autophagy-related protein 7 mRNA (77). This impairs macrophage autophagy (78), reducing the degradation of ox-LDL and promoting foam cell formation.

Furthermore, EVs can promote foam cell formation by influencing macrophages polarization. M1 macrophages, characterized by high expression of scavenger receptors such as SR-A and CD36 (79), exhibit reduced expression of ABCA1 and ABCG1, leading to enhance lipid uptake and impair efflux, thereby promoting cholesterol accumulation and foam cells transformation. EVs from different sources influence this polarization through specific mechanisms. Under oxLDL stimulation, endothelial cells secrete EVs rich in miR-126, which, after being taken by macrophages, upregulate M1 polarization-related genes such as inducible nitric oxide synthase(NOS2) and interleukin-12(IL-12) (80) through pathways involving phosphatase and tensin homolog(PTEN), PI3K/AKT, and NF-κB (81). The NO produced by NOS2 (82), not only promotes inflammation response but also reacts with ROS to generate peroxynitrite (83), exacerbating local tissue damage and creating a vicious cycle. In addition, platelet-derived EVs containing platelet-derived growth factor(PDGF) can activate the RAS/ERK pathway by binding to PDGF receptors on macrophages (84), regulatin transcription factors such as activator protein 1(AP-1) and cAMP response element-binding protein (85), thereby enhancing the expression of pro-inflammatory genes such as TNF-α and IL-12 (86). PDGF can also promotes macrophage proliferation and migration (87), further amplifying the local inflammatory response.

### 3.3 EVs affect plaque stability

EVs not only participate in lipid metabolism but also influence plaque stability by mediating inflammatory responses, regulating vascular smooth muscle cell(VSMC) proliferation, migration, and phenotypic transformation, and promoting AS plaque calcification (Figure 1).

During AS progression, inflammatory cells such as macrophages and T lymphocytes accumulate within plaques and release EVs enriched with specific cytokines (88). T lymphocyte-derived EVs carry pro-inflammatory factors like interferon-gamma (IFN-*γ*) (89), which inhibit cholesterol efflux, promote foam cell formation, and contribute to lipid core expansion (90). In advanced plaques, inflammatory cell-derived EVs are rich in matrix metalloproteinases (MMPs) (91), such as MMP-2 and MMP-9, which degrade extracellular matrix components like collagen and elastin (92), thinning the fibrous cap and increasing the risk of rupture.

EVs from endothelial cells and platelets influence plaque structure by regulating VSMC behavior. Endothelial cell-derived EVs contain platelet-derived growth factor (PDGF) and transforming growth factor beta (TGF-β), which act via distinct pathways. PDGF activates the PI3K-Akt pathway to upregulate Cyclin D1 expression (93, 94), promoting G1/S phase transition and VSMC proliferation (95). TGF-β activates the Smad pathway, inducing phenotypic switching of VSMCs from contractile to synthetic states (96, 97). These synthetic VSMCs secrete more type I collagen, reduce elastin content, and upregulate MMP-9 (98), thereby weakening the fibrous cap (99). Platelet-derived EVs carry chemokines such as monocyte chemoattractant protein-1 (MCP-1) (100), which bind to C-C motif chemokine receptor 2 (CCR2) on VSMCs and activate G protein-coupled signaling to promote migration (101).

EVs also contribute to plaque calcification via bone morphogenetic proteins (BMPs) and miR-221/222. BMP-2 binds to BMP receptors on VSMCs and activates Smad signaling (102), inducing osteogenic transcription factors such as runt-related transcription factor 2 and Osterix (103), leading to calcium deposition (104). EVs enriched in miR-221/222 enhance VSMC proliferation, migration, and phenotypic switching (105), and may regulate phosphate metabolism by modulating ectonucleotide pyrophosphatase/phosphodiesterase 1 and phosphate transporter 1 (106), thereby promoting calcification (107).

Endothelial-VSMC communication also relies on EVs. Damaged or inflamed endothelial cells release EVs containing PDGF (108), which bind to receptors on VSMCs and activate the mitogen-activated protein kinase (MAPK) signaling pathway. ERK1/2 translocates into the nucleus, phosphorylates transcription factors such as Ets-like transcription factor 1 (Elk-1), and enhances the expression of FBJ murine osteosarcoma viral oncogene homolog (c-Fos) and Jun proto-oncogene (c-Jun), forming the AP-1 complex (109, 110). This complex promotes transcription of genes such as Cyclin D1 (111) and MMPs (112), facilitating VSMC proliferation, extracellular matrix degradation, and plaque progression. In addition, miR-21 from endothelial-derived EVs (113) suppresses programmed cell death protein 4 (114), reducing MMP inhibition and further impairing plaque stability.

Macrophage-VSMC communication is another critical axis. Macrophages exposed to ox-LDL secrete EVs containing chemokines such as MCP-1 (115), which activate the PI3K-Akt signaling pathway upon uptake by VSMCs (116). This enhances pseudopodia formation and promotes VSMC migration (117). Meanwhile, the PI3K-Akt pathway also upregulates MMP-9, facilitating elastin degradation, VSMC infiltration into the intima, and AS plaque development (118, 119).

### 3.4 The role of EVs in thrombosis

EVs play an important role in thrombosis by promoting platelet activation and aggregation, and regulating coagulation cascade (Figure 1). In inflammatory or thrombotic micro-environments, P-selectin on endothelial cell-derived EVs bind to P-selectin glycoprotein ligand-1 (PSGL-1) on platelets, triggering their transformation from a resting discoid shape to an activated state with pseudopod (120). Activated platelets and erythrocytes (121) release EVs enriched in phosphatidylserine (PS) (122), which, along with thrombin carried by EVs, can activate platelets (123) via protease-activated receptors protease-activated receptor 1(PAR1) and protease-activated receptor 4(PAR4), initiating the phospholipase C (PLC) pathway (124, 125). PLC promotes inositol 1,4,5-trisphosphate(IP3), leading to calcium release from the endoplasmic reticulum and increasing intracellular ca^2+^ levels, enhancing platelet activation (126).

EVs also deliver pro-aggregatory factors. ADP within P-EVs (127) bind to P2Y purinoceptor 1 (P2Y1) and P2Y purinoceptor 12 (P2Y12) receptors (128) on the platelets, activating the PLC-protein kinase C (PKC) signaling pathway. This cascade further elevates intracellular ca^2+^, induces shape changes and activates fibrinogen receptors (glycoprotein IIb/IIIa complex), promoting platelet aggregation (129).

TF (Tissue factor)-positive EVs are central to initiating the coagulation During vascular inflammation (130, 131), activated monocytes release a large number of TF-rich MDEVs (40, 132). In circulation, TF on MDEVs (133) binds to factor VII, forming a TF-VIIa complex (134) that activates coagulation factor X,which can promote the release of endothelial cell-derived TF + EVs (135), and triggers the extrinsic coagulation pathway (136). However, activated coagulation factor VII can also play a protective role by inducing endothelial cells to secrete EVs rich in miR-10a through the activated Factor VII-endothelial protein C receptor-protease activated receptor 1(FVIIa-EPCR-PAR1) axis (137). After being taken up by monocytes, these EVs can downregulate the transforming growth factor-β-activated kinase 1 pro-inflammatory signaling pathway, creating an anti-inflammatory environment (138). In addition, tumor cell-derived TF + EVs can also promote thrombosis (139). Concurrently, PS exposure on the EV surface facilitates the assembly of the prothrombinase complex, which effectively converts prothrombin to thrombin (140, 141), promoting the release of EVs rich in pro-coagulation proteins and adhesion proteins from platelets (142) and amplying coagulation.On the contrary, EVs derived from endothelial cells and leukocytes carry plasmin and plasminogen activators, playing a comprehensive role in regulating thrombus balance (143).

## 4 Evs in ASCVD diagnosis and treatment

### 4.1 EVs as biomarkers

**Figure 2:**
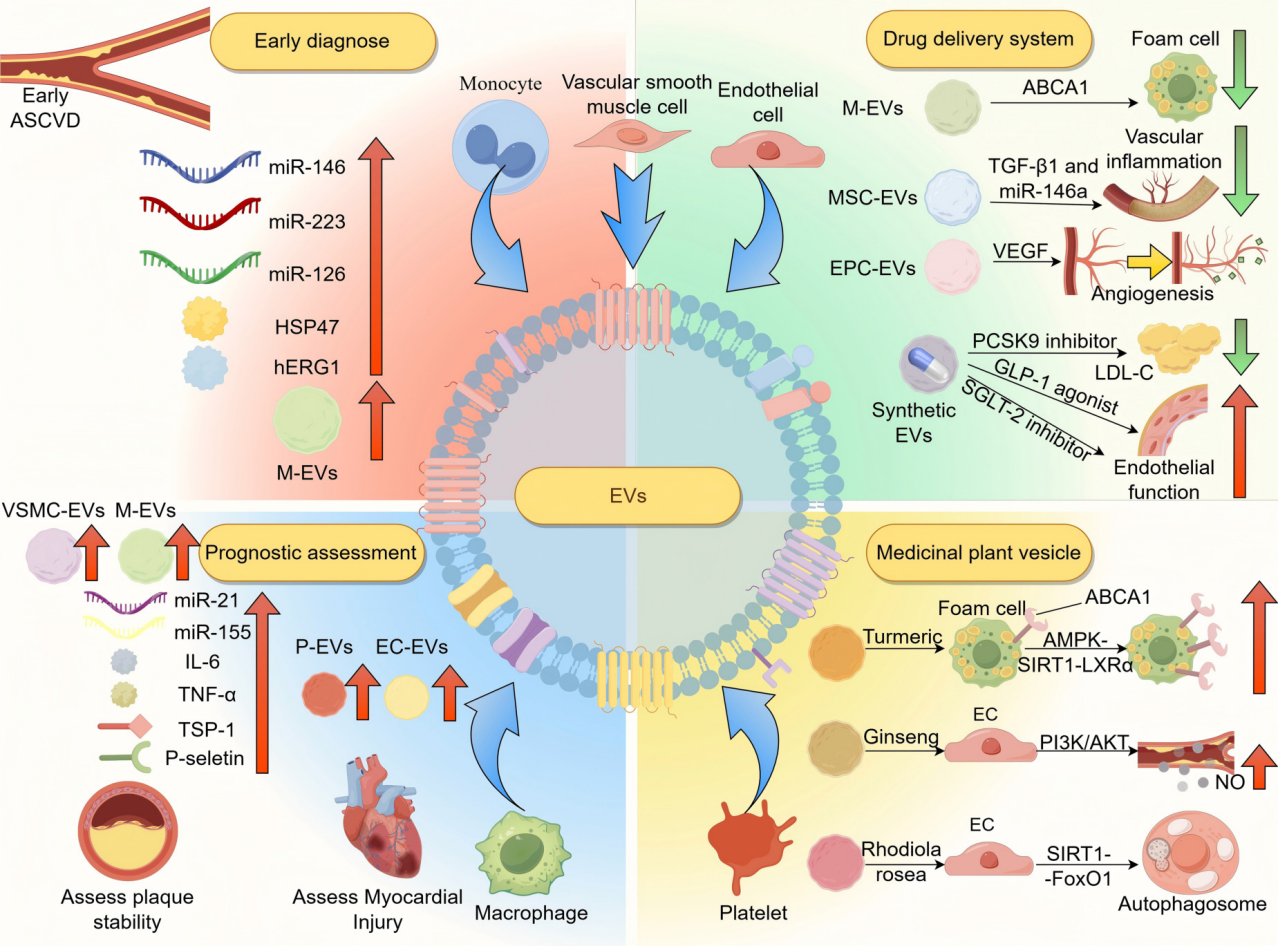
The application of EVs in the diagnosis and treatment of ASCVD. EVs serve as diagnostic markers in ASCVD. M-EVs and EVs enriched with miR-146a, miR-223, and miR-126 are elevated in early atherosclerosis. Hsp47 and hERG1-related changes reflect cardiac stress and arrhythmia risk. EV quantity and origin vary by stage: SMC- and macrophage-derived EVs increase during plaque formation, while platelet- and endothelial-derived EVs rise in ACS. EVs from severe cases show elevated IL-6, TNF-α, TSP-1, P-selectin, miR-21, and miR-155. Therapeutically, EVs can deliver ABCA1, miR-146a, TGF-β1, VEGF, PCSK9 inhibitors, SGLT2 inhibitors, and GLP-1 receptor agonists to reduce inflammation, promote angiogenesis, and improve endothelial function. Plant-derived EVs activate AMPK-SIRT1-LXRα, PI3K/Akt, and SIRT1-FoxO1 pathways to enhance endothelial repair and autophagy. ASCVD, atherosclerotic cardiovascular disease; Hsp47, heat shock protein 47; hERG1, human ether-à-go-go-related gene 1; M-EVs, macrophage-EVs; VSMC-EVs, vascular smooth muscle cell-EVs; IL-6, interleukin-1; TNF-α, tumor necrosis factor-alpha; TSP-1, thrombus proteins like thrombospondin-1; P-EVs, platelet-EVs; EC-EVs, endothelial-derived EVs; MSC-EVs, mesenchymal stem cell-EVs; EPC-EVs, endothelial progenitor cell-EVs; ABCA1, ATP-binding cassette transporter A1; TGF-β1, transforming growth factor beta 1; VEGF, vascular endothelial growth factor; PCSK9, proprotein convertase subtilisin/kexin type 9; AMPK-SIRT1-LXRα, AMP-activated protein kinase-sirtuin 1-liver X receptor alpha; PI3K/Akt, phosphatidylinositol 3-kinase/protein kinase B; SIRT1-FoxO1, sirtuin 1-forkhead box protein O1.

In recent years, EVs have shown increasing value in the early diagnosis of cardiovascular diseases like ASCVD, as well as in monitoring disease progression, evaluating prognosis, and assessing treatment response (Figure 2; Table 1).

**Table 1:** Summary of the diagnosis and treatment of EVs in ASCVD.

Category	Application	Key components/mechanisms	Function/outcome
EVs as Biomarkers	Early Diagnosis	Monocyte-derived EVs (elevated in early AS)	Promote endothelial dysfunction, thrombosis (149)
		miR-146a, miR-223, miR-126 (dysregulated in EVs) (147)	miR-146a-5p↓: M1 macrophage activation↑; miR-223↓: myocardial fibrosis↑ (152)
		Hsp47, hERG1 (EV proteins) (155)	Hsp47↑: cardiac fibrosis; hERG1 dysfunction: arrhythmias (156)
	Prognostic Assessment	VSMC/macrophage EVs↑ (plaque formation) (157)	Reflect plaque stability (157)
		Platelet/endothelial EVs↑ (ACS) (157)	Correlate with myocardial injury, cardiovascular risk (158)
	Treatment Monitoring	CD14+/CD41+ EVs↓ (post-statin therapy) (166)	Anti-inflammatory, antithrombotic effects (167)
EVs as Drug Carriers	Anti-inflammatory Therapy	MSC-derived EVs (miR-146a, TGF-β1) (182)	Suppress NF-κB signaling, reduce plaque inflammation (184)
	Cholesterol Regulation	Macrophage EVs (ABCA1) (180, 181)	Enhance cholesterol efflux in foam cells (180, 181)
	Angiogenesis	EPC-derived EVs (VEGF) (185)	Promote endothelial proliferation, improve perfusion (185)
Synthetic EVs	Lipid Metabolism	PCSK9 inhibitor-loaded EVs (191)	LDL receptor ↑, promote LDL-C clearance (191)
	Vascular Homeostasis	NO-releasing EVs (192)	Improve endothelial function (192)
	Immune Modulation	Anti-PD-L1 antibody-loaded EVs (195, 196)	Block PD-1/PD-L1 pathway, enhance T cell activity (195, 196)
Plant-Derived Vesicles	Cholesterol Efflux	Curcumin EVs (AMPK-SIRT1-LXRα pathway) (204)	ABCA1↑, inhibit NLRP3 inflammasome (205)
	Anti-Oxidative Stress	Rhodiola EVs (salidroside) (208)	Activate SIRT1-FoxO1 autophagy, oxidative damage↓ (209)
	Anti-Inflammatory	Ginseng EVs (ginsenosides) (207)	Activate PI3K/Akt, eNOS phosphorylation↑, NF-κB↓ (207)

EVs, extracellular vesicles; SR-A, scavenger receptor class A; CD-36, cluster of differentiation 14; Fas/FasL, tumor necrosis factor receptor superfamily member 6/tumor necrosis factor ligand superfamily member 6; TSP-1, thrombus proteins like thrombospondin-1; PI3K/Akt, phosphatidylinositol 3-Kinase/protein kinase B; SR-BI, scavenger receptor class B type I; TLR4, toll-like receptor 4; PLC-PKC, phospholipase C-protein kinase C; P2Y1, P2Y purinoceptor 1; P2Y12, P2Y purinoceptor 12; PAR1, protease activated receptor 1; PAR4, protease activated receptor 4; PS, phosphatidylserine; PDGF, platelet-derived growth factor; TGF-β, transforming growth factor beta; PSGL-1, P-selectin glycoprotein ligand-1; MCP-1, monocyte chemoattractant protein-1; CCR2, C-C motif chemokine receptor 2.

Studies indicates that specific EVs subpopulations and their bioactive components could be potential early diagnostic markers or indicators of disease progression (144). For example, monocytes-derived EVs are significantly elevated in the blood of patients with early AS (145), which may contribute to endothelial dysfunction. This plays a role in ASCVD initiation and progression (12). IL-33 can induce a significant increase in TF + EVs derived from monocytes, promoting thrombosis, suggesting that IL-33 may serve as a biomarker for predicting ASCVD (146). The expression levels of miR-146a, miR-223, and miR-126 in EVs change significantly in AS patients (147). miR-126, which is abundant in endothelial-derived EVs (148), plays a protective role. Reduced miR-126 increases Spred-1, blocks VEGF signaling, and impairs endothelial function, promoting ASCVD (149). miR-146a-5p derived from cardiomyocyte-derived EVs can inhibit M1 macrophage activation and reduce inflammatory responses by targeting CD80 (150) and TNF receptor-associated factor 6 (151). Therefore, the expression of miR-146a-5p in cardiomyocyte-derived EVs from ST-segment elevation myocardial infarction patients is significantly reduced (152). miR-223 is highly expressed in monocyte-derived EVs and can alleviate myocardial inflammation by targeting semaphorin 3A and signal transducer and activator of transcription 3 (153). Therefore, miR-223 is significantly reduced in EVs from patients with heart failure due to inflammation-induced myocardial fibrosis (154). EV proteins like heat shock protein 47 (Hsp47) and human ether-à-go-go-related gene 1 (hERG1) also aid early diagnosis—Hsp47 reflects cardiac stress and fibrosis (155), while hERG1 dysfunction may cause arrhythmias (156).

EVs are also valuable for prognostic assessment. During early plaque formation, levels of EVs derived from VSMCs and macrophages significantly increases (157), potentially reflecting plaque stability. Meanwhile, in acute coronary syndrome, EVs from platelets and endothelial cells increase sharply, correlating with myocardial injury and the risk of cardiovascular events (158). Moreover, EVs composition Reflects disease severity: inflammatory cytokines such as IL-6 and TNF-α, TSP-1 and P-selectin (159), and miRNAs such as miR-21, miR-155 (160) and miR-133 (161), are evaluated in patients with severe ASCVD. miR-21 and miR-155 in EVs enhance inflammation in AS plaques and worsen prognosis, making them potential biomarkers for disease progression and outcome prediction.In the MINERVA study, researchers conducted a retrospective case-control analysis of 269 patients with acute chest pain and found that low levels of the plasma EVs protein Cystatin C in patients with low levels of high-sensitivity cardiac troponin were associated with unstable angina (162, 163). This suggests that EVs may be useful in the risk stratification of cardiovascular events. Data analysis from the Athero-Express biobank showed that among 864 patients undergoing carotid endarterectomy, preoperative levels of EV-related proteins (such as CD14 and Cystatin C) were significantly associated with the risk of major cardiovascular events within three years after surgery (164, 165). This indicates that EV-derived proteins could serve as biomarkers for assessing remaining cardiovascular risk and may help identify high-risk patients for more tailored secondary prevention.

EVs also have potential in motoring treatment responses. In patients with AMI receiving statin therapy, levels of monocyte-derived CD14^+^ EVs and platelet-derived CD41^+^ EVs significantly decreased post-treatment (166), reflecting the anti-inflammatory and antithrombotic effects of statins and serving as markers of therapeutic efficacy (167). In patients with AMI after PCI who were treated with the P2Y12 antagonist ticagrelor, the concentrations of plasma platelet-derived EVs, endothelial cell-derived EVs, leukocyte-derived EVs, fibrinogen-exposed EVs, and PS-exposed EVs all significantly decreased (168), indicating that this regimen has good anti-inflammatory and antithrombotic effects (169). Furthermore, Changes in inflammatory-related EVs content may indicate treatment tolerance (170), helping guide timely therapeutic adjustments and improving clinical decision-making.

### 4.2 EVs as drug delivery systems

The therapeutic value of EVs lies in their role as intervention targets and drug delivery vehicles (Figure 2; Table 1). During AS progression, EVs affect the disease progression in several ways, such as regulating lipid metabolism, inflammation and endothelial function (12). Therefore, regulating their production or altering their cargo offers new approaches for ASCVD treatment.

Recent researches indicates that natural IgM antibodies may inhibit thrombosis by competing with coagulation factor X/Xa for binding to coagulation-related EVs (171). Exosomes derived from adipose-derived mesenchymal stem cells can significantly reduce the expression of pro-inflammatory cytokines such as TNF-α and IL-6 (172). This anti-inflammatory effect is mainly attributed to the transfer of miR-21 and miR-146a, which inhibit TLR4/NF-κB signaling in macrophages, thereby suppressing M1 polarization and reducing the production of pro-inflammatory mediators. These exosomes also significantly improve cardiac function after myocardial infarction by modulating macrophage phenotypes and reducing myocardial fibrosis and inflammatory cell infiltration (173). Exosomes derived from umbilical cord mesenchymal stem cells are rich in miR-29a-3p, which can activate the VEGF signaling pathway (174), thereby enhancing the proliferation and migration of endothelial cells and promoting angiogenesis (175). Mechanistically, miR-29a-3p targets PTEN and upregulates the PI3K/Akt/eNOS axis, increasing NO production and supporting vascular homeostasis.

As drug carriers, EVs have distinct advantages: they exhibit good biocompatibility and low immunogenicity (176), enabling them to evade immune clearance. Their surface molecules can mediate targeted delivery to specific cells (177), and their lipid bilayer protects encapsulated drugs from degradation (178, 179), allowing efficient release via membrane fusion (167).

Therapeutically, EVs are involved in key ASCVD processes. Macrophage-derived EVs carrying ABCA1 promote cholesterol efflux from foam cells by enhancing reverse cholesterol transport pathways (180, 181). This process helps stabilize plaques and prevent necrotic core expansion. Mesenchymal stem cell-derived EVs loaded with miR-146a and TGF-β1 (182) can target atherosclerotic plaques, suppress NF-κB signaling (183), reduce vascular inflammation, and regulate VSMC phenotype by inhibiting osteogenic transition and promoting contractile markers (184). Furthermore, EPC-derived EVs enriched with VEGF promote angiogenesis and improve tissue perfusion in ischemic myocardium (185). These EVs activate VEGFR2 on endothelial cells and downstream PI3K/Akt signaling, which enhances endothelial proliferation, migration, and capillary network formation (186).

### 4.3 Application of synthetic EVs

Artificially synthesized EVs can mimic natural EVs by carrying therapeutic molecules and targeting specific tissues (Figure 2 and Table 1). Compared with natural EVs, synthetic EVs allow for improved surface modification to enhance targeting and drug-loading capacity and their profucing yields higher purity with reduced batch variability (187), addressing limitations in the clinical applications of natural EVs (188). Moreover, emerging light-responsive EVs have been developed, which enable spatiotemporal control of drug release upon specific light stimulation, thus improving delivery precision and minimizing off-target toxicity (189, 190).

In the treatment of ASCVD, synthetic EVs show broad potential across multiple pathological mechanisms. To regulate lipid metabolism, synthetic EVs can deliver PCSK9 inhibitors, which increase hepatic LDL receptor levels and promote LDL-C clearance (191). By encapsulating these agents in EV-mimetic nanocarriers, hepatic uptake is enhanced and systemic side effects reduced. To improve vascular homeostasis, synthetic EVs have been designed to release nitric oxide (NO), which activates the soluble guanylate cyclase pathway and promotes vasodilation (192).

Furthermore, synthetic EVs can encapsulate SGLT2 inhibitors or GLP-1 receptor agonists to improve endothelial function and glycemic control in patients with metabolic syndrome (193). Their surface can be functionalized with endothelial-targeting peptides (e.g., RGD motifs) to enhance specificity and accumulation in vascular lesions. Synthetic EVs can also carry VEGF to stimulate angiogenesis through VEGFR2-mediated PI3K/Akt/eNOS signaling (194), or transport anti–PD-L1 antibodies to block the PD-1/PD-L1 pathway, thus enhancing T-cell activation and restoring immune balance within the plaque microenvironment (195, 196).

In terms of inflammation regulation, artificially synthesized EVs loaded with miR-146a can significantly reduce inflammatory factors and inhibit the polarization of M1 macrophages (197) by intervening in the TLR4/NF-κB pathway (198). In maintaining plaque stability, artificially synthesized EVs can deliver tissue inhibitor of metalloproteinases 3 mRNA, suppress the expression of MMPs, and reduce collagen degradation (199).

However, synthetic EVs may still be cleared by the immune system or bind to non-target cells during circulation. To improve targeting accuracy, it may be necessary to develop multi- ligand surface modifications that recognize several key targets, reducing off-target effects and enhancing therapeutic efficacy.

### 4.4 Applications of medicinal plant vesicles

Medicinal plant vesicles, due to their natural origin, show unique therapeutic potential in treating ASCVD by regulating lipid metabolism, reducing oxidative stress and inflammation, and promoting angiogenesis (200) (Figure 2 and Table 1). They offer strong targeting ability (201) and excellent biocompatibility (202), making them less likely to trigger immune responses compared to synthetic carriers. Recent studies have also found that microsphere systems using poly(lactic-co-glycolic acid) (PLGA) as a carrier can achieve sustained release *in vivo*, prolonging the duration of drug action and reducing the frequency of administration for patients (203). In the future, it may be considered to apply PLGA to load medicinal plant vesicles, extending the *in vivo* circulation time of the plant vesicles through the sustained release characteristics of PLGA.

Curcumin-derived EVs (204), for instance, activate the AMPK-SIRT1-LXRα pathway in foam cells, upregulating ABCA1 and enhancing cholesterol efflux (205). They also directly bind and inhibit the NLRP3 inflammasome, thereby attenuating the downstream release of IL-1β and suppressing vascular inflammation associated with atherosclerosis progression (206). Ginseng-derived EVs, enriched in ginsenosides, promote eNOS phosphorylation and NO production through PI3K/Akt pathway activation while inhibiting NF-κB–mediated inflammatory gene transcription via SIRT1 modulation (207). Rhodiola-derived EVs, containing salidroside (208), activate the sirtuin 1-forkhead box O1(SIRT1-FoxO1) to induce autophagy and reduce oxidative damage in endothelial cells, while also promoting ABCA1 and inhibiting ox-LDL receptor expression to reduce foam cell formation. Despite their promise and good biocompatibility (209), further studies are needed to assess their safety and potential side effects.

## 5 Limitations of EVs in the clinical application of ASCVD

Although EVs have shown great promise in ASCVD, their clinical application still faces considerable challenges. As biomarkers, their utility is hindered by technical limitations in isolation and purification. EVs are typically present in low abundance in body fluids (210), and conventional isolation methods such as ultracentrifugation, precipitation, and immunocapture often suffer from drawbacks including prolonged processing time, high cost, sample loss, and compromised EV integrity (211, 212). Moreover, these methods frequently co-isolate contaminants from other cellular components, which can reduce the specificity of EV detection and introduce false-positive or false-negative results in biomarker analysis (213, 214).

It is important to note that the impact of purity varies depending on the intended application of EVs. When EVs are studied as mediators of intercellular communication or therapeutic agents, the presence of non-vesicular contaminants may significantly distort functional interpretations. However, in the context of EVs as biomarkers, minor contamination may not critically impair their diagnostic value, provided that marker-specific signatures are preserved.

To address purification challenges, newer methods such as size-exclusion chromatography (SEC) have gained attention. SEC allows for the gentle separation of EVs from complex biofluids by physical exclusion, avoiding the use of harsh mechanical or chemical conditions that could damage vesicle integrity or surface proteins (215, 216). This approach improves EV purity while maintaining their biological functionality, which is crucial for downstream diagnostic and therapeutic applications. Nonetheless, SEC also has limitations. It is not very effective in removing certain high-density protein aggregates or lipoproteins, which may still interfere with subsequent analyses (217).

In terms of therapeutic applications, long-term efficacy and safety data on EV-based treatments remain limited. Challenges such as low yield, suboptimal purity, and potential functional degradation of EVs during processing continue to hinder their clinical translation (218). Therefore, overcoming these barriers will require the development of innovative isolation techniques, standardized quality control frameworks, and rigorous preclinical and clinical studies to fully realize the diagnostic and therapeutic potential of EVs in ASCVD.

## 6 Summary and outlook

As important mediators of intercellular communication, EVs play multifaceted roles in the development of ASCVD. This review summarizes the biological characteristics of EVs and their involvement in the initiation and progression of ASCVD. Clinically, EVs act as emerging biomarkers for early diagnosis and prognosis assessment. Their natural targeting ability and biocompatibility also make them promising drug delivery vehicles, with engineered and plant-based EVs offering new personalized therapy.

Future research should integrate multi-omics technologies to track dynamic changes in EV components, establish standardized isolation and identification protocals, and validate therapeutic strategies in preclinical models. Further exploration of EVs interactions with metabolic and immune system, along with AI-based prediction models, may open new ideas for precision medicine.
